# Replication kinetics and persistence of a conditionally live attenuated SIV (SIVrtTA) *in vivo* confers protection against homologous and heterologous wild-type challenge in macaques

**DOI:** 10.1186/1742-4690-10-S1-P5

**Published:** 2013-09-19

**Authors:** Neil Berry, Maria Manoussaka, Claire Ham, Deborah Ferguson, Mark Page, Richard Stebbings, Atze Das, Martin Cranage, Neil Almond, Ben Berkhout

**Affiliations:** 1 Virology, NIBSC, South Mimms, Hertfordshire, UK; 2 Laboratory of Experimental Virology, Department of Medical Microbiology, Centre for Infection and Immunity Amsterdam, Academic Medical Center of the University of Amsterdam, Amsterdam, The Netherlands; 3 Division of Cellular and Molecular Medicine, St. George’s, University of London, London, UK

## Background

Vaccination with live attenuated SIV confers potent protection against wild-type SIV challenge in the SIV/macaque model of HIV. Although safety concerns preclude direct application of this vaccine approach in humans, a clearer understanding of the mechanistic processes at play may lead to better HIV vaccines. A novel live attenuated SIVmac239Δnef vaccine (SIVrtTA), dependent on doxycycline (dox) administration for replication, has been evaluated for its ability to protect *in vivo* against independent homologous and heterologous pathogenic wild-type (wt) SIV challenges, and the role of vaccine persistence assessed. We recently demonstrated immunophenotyping of SIVrtTA vaccinees prior to wt challenge drives global polarisation of a T cell memory phenotype, particularly in the CD4 population, toward an effector memory response.

## Materials and methods

Initially, two groups of six Indian rhesus macaques (RM) were vaccinated with SIVrtTA with an oral doxycycline dosing regime for 6 months. In Group A, doxycycline was stopped 8 weeks before homologous wild-type SIVmac239 challenge but continued in Group B. Separately, Mauritian cynomolgus macaques (CM) were vaccinated with SIVrtTA in three groups (Grp A; 20 wks dox-on, Grp B; 3 wks dox-on, 17 wks dox-off; Grp C; 3 wks dox-on) and challenged with the heterologous wild-type SIVsmE660. Kinetics of vaccine virus replication and superinfection resistance outcomes were determined by sensitive and specific molecular and virological assays and detailed immunological studies performed.

## Results

Following wild-type challenge with either SIVmac239 or SIVsmE660, all SIVrtTA vaccinees exhibited significantly lower levels of breakthrough virus which in 2/8 SIVrtTA vaccinees challenged with SIVmac239 and 10/18 challenged with SIVsmE660 were protected from detectable wt virus. While the kinetics of vaccine virus replication differed between species, in Indian RM enhanced control of wild-type virus was correlated with a transitory shoulder of sustained plasma viral RNA load in the period up to 100 days post-vaccine delivery. On the day of challenge, a high frequency of circulating CD8+ T_EM_ was associated with protection but was not necessarily essential. In Mauritian CM there was a clear correlation between persistence of the vaccine virus and protection with 6/6 protected against detectable SIVsmE660 infection after 20 weeks vaccination (dox ‘on’), with 1/6 protected after 3 weeks vaccination (dox ‘on’) and 3/6 dox ‘on’ for 3 weeks followed by 17 weeks dox ‘off’.

## Conclusions

Together, these data suggest a maturation of protection with longer periods of vaccination is beneficial, even when the vaccine-virus is apparently ‘switched-off’. Since the genetics and antigenic relatedness of vaccine and challenge virus does not appear to predict the outcome of these vaccine studies, the kinetics and relative persistence of vaccine virus replication in contributing to adaptive and innate immune responses are being evaluated. The outcome of these studies will inform rational HIV/AIDS vaccine design

